# Bis(quinolin-8-ol)silver(I) 2-hydr­oxy-3,5-dinitro­benzoate

**DOI:** 10.1107/S1600536809045905

**Published:** 2009-11-07

**Authors:** Chun-Lan Zhang, Fang-Fang Jian

**Affiliations:** a Microscale Science Institute, Biology Department, Weifang University, Weifang 261061, People’s Republic of China; b Microscale Science Institute, Weifang University, Weifang 261061, People’s Republic of China

## Abstract

The title compound, [Ag(C_9_H_7_NO)_2_](C_7_H_3_N_2_O_7_), was prepared from 3,5-dinitro­salicylic acid (DNS), quinolin-8-ol and AgNO_3_. The Ag^I^ atom is coordinated by two N atoms and two O atoms from two quinolin-8-ols in a roughly planar [maximum deviation = 0.223 (2) Å] environment. The two quinolin-8-ol ligands are bent slightly with respect to each other, making a dihedral angle of 9.55 (9)°. The DNS anion inter­acts with the silver complex through O—H⋯O hydrogen bonds

## Related literature

For related structures, see: Smith & Thomasson (1999 ▶); Smith *et al.* (2001 ▶); Wu *et al.* (2006 ▶).
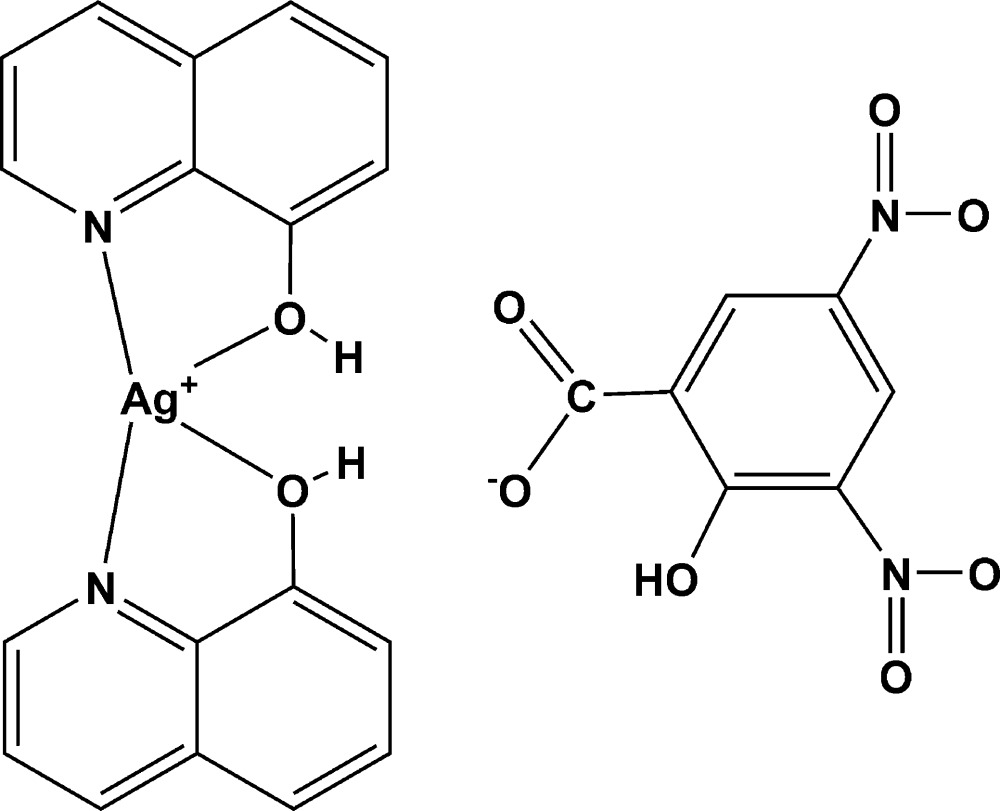


## Experimental

### 

#### Crystal data

[Ag(C_9_H_7_NO)_2_](C_7_H_3_N_2_O_7_)
*M* *_r_* = 625.30
Monoclinic, 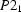

*a* = 9.0154 (18) Å
*b* = 7.6122 (15) Å
*c* = 17.138 (3) Å
β = 104.38 (3)°
*V* = 1139.3 (4) Å^3^
*Z* = 2
Mo *K*α radiation
μ = 0.95 mm^−1^
*T* = 293 K
0.20 × 0.15 × 0.11 mm

#### Data collection

Bruker SMART CCD area-detector diffractometer
Absorption correction: none
10841 measured reflections
4602 independent reflections
4356 reflections with *I* > 2σ(*I*)
*R*_int_ = 0.022

#### Refinement

*R*[*F* ^2^ > 2σ(*F* ^2^)] = 0.024
*wR*(*F* ^2^) = 0.057
*S* = 1.09
4602 reflections
353 parameters
1 restraint
H-atom parameters constrained
Δρ_max_ = 0.70 e Å^−3^
Δρ_min_ = −0.30 e Å^−3^
Absolute structure: Flack (1983 ▶), 1770 Friedel pairs
Flack parameter: 0.006 (18)

### 

Data collection: *SMART* (Bruker, 1997 ▶); cell refinement: *SAINT* (Bruker, 1997 ▶); data reduction: *SAINT*; program(s) used to solve structure: *SHELXS97* (Sheldrick, 2008 ▶); program(s) used to refine structure: *SHELXL97* (Sheldrick, 2008 ▶); molecular graphics: *ORTEPIII* (Burnett & Johnson, 1996 ▶) and *ORTEP-3 for Windows* (Farrugia, 1997 ▶); software used to prepare material for publication: *SHELXL97*.

## Supplementary Material

Crystal structure: contains datablocks global, I. DOI: 10.1107/S1600536809045905/dn2508sup1.cif

Structure factors: contains datablocks I. DOI: 10.1107/S1600536809045905/dn2508Isup2.hkl

Additional supplementary materials:  crystallographic information; 3D view; checkCIF report

## Figures and Tables

**Table 1:** Hydrogen-bond geometry (Å, °)

*D*—H⋯*A*	*D*—H	H⋯*A*	*D*⋯*A*	*D*—H⋯*A*
O1—H1*AA*⋯O8	1.00	1.60	2.602 (3)	175
O2—H2*AA*⋯O9	0.77	1.88	2.636 (3)	168
O3—H3*B*⋯O9	0.82	1.74	2.483 (3)	150

**Fig. 1.**
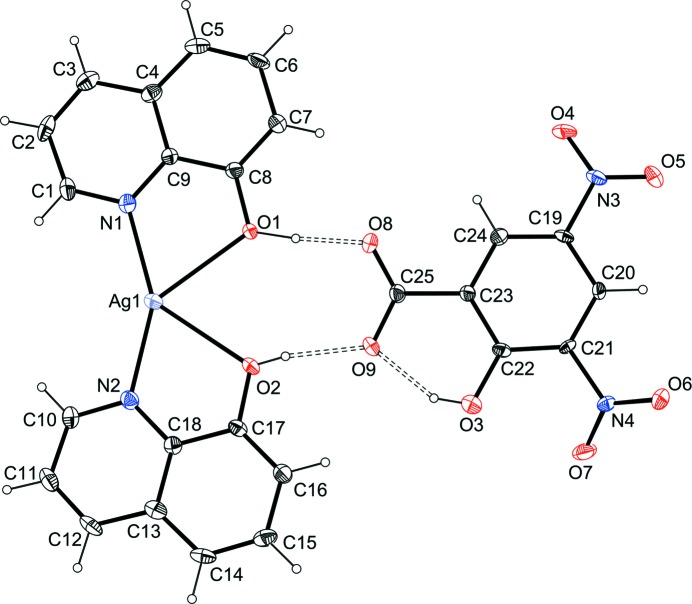
The molecular structure of the title compound with the atom-labeling scheme. Displacement ellipsoids are drawn at the 30% probability level. H atoms are represented as small spheres of arbitrary radii. Hydrogen bonds are shown as dashed lines.

## supplementary crystallographic information

### Comment

Quinolin-8-ol [quinolin-8-ol (oxine)] is well known as a particularly versatile ligand for use in metal complex chemistry (G. Smith, *et al.*,2001). It is also known that most of Ag^I^ in biological systems is not in the form of free Ag^I^ ions, but is coordinated by the abundance of biological ligands (Wu, *et al.*,2006). As part of our search for new biologically active compounds the title compound has been synthesized and we report its crystal structure here.

Scheme I

The Ag^I^ atom is coordinated by two N atoms and two O atoms from two quinolin-8-ols in a roughly planar environment with the largest deviation from the mean plane of the non H atoms being 0.223 (2)Å at C14 (Fig. 1). However, the two quinolin-8-ols are slightly bent with respect to each other making a dihedral angle of 9.55 (9)°. In the DNS anion, the NO_2_ and CO_2_ groups are twisted with respect to the phenyl ring making dihedral angles of of 29.5 (1)° for C21, N4, O6, O7, 10.7 (2)° for C19, N3, O4, 05 and 10.0 (2)° for C23, C25, O8, O9. All of the bond lengths and bond angles are in normal ranges (Smith, *et al.*,1999; Smith, *et al.*,2001; Wu, *et al.*, 2006).

There are O—H···O hydrogen-bond interactions between two quinolin-8-ol and DNS which stabilize the crystal structure (Table 1, Fig. 1).

### Experimental

The title compound(I) was prepared by the process as following: A mixture of 3,5-Dinitrosalicylic acid (0.01 mol), salt of quinolin-8-ol and sulfuric acid (0.02 mol) was stirred in distilled water (30 ml) for 3 h to obtain yellow deposit. A mixture of the deposit and AgNO3(0.01 mol) was stirred in ethanol (20 ml) at 353 K for 5 h, then afford the title compound (yield 83%). Single crystals suitable for X-ray measurements were obtailed by recrystallization from ethanol at room temperature.

### Refinement

H atoms were included in calculated positions, with C—H distances constrained to 0.93Å (aromatic CH) and O—H distances constrained to 0.86Å and with *U*_iso_=1.2–1.5*U*_eq_.

### Crystal data

**Table:**

[Ag(C_9_H_7_NO)_2_](C_7_H_3_N_2_O_7_)	*F*(000) = 628
*M**_r_* = 625.30	*D*_x_ = 1.823 Mg m^−^^3^
Monoclinic, *P*2_1_	Mo *K*α radiation, λ = 0.71073 Å
Hall symbol: P 2yb	Cell parameters from 4356 reflections
*a* = 9.0154 (18) Å	θ = 3.6–27.6°
*b* = 7.6122 (15) Å	µ = 0.95 mm^−^^1^
*c* = 17.138 (3) Å	*T* = 293 K
β = 104.38 (3)°	Block, yellow
*V* = 1139.3 (4) Å^3^	0.20 × 0.15 × 0.11 mm
*Z* = 2	

### Data collection

**Table:**

Bruker SMART CCD area-detector diffractometer	4356 reflections with *I* > 2σ(*I*)
Radiation source: fine-focus sealed tube	*R*_int_ = 0.022
graphite	θ_max_ = 27.6°, θ_min_ = 3.6°
φ and ω scans	*h* = −11→11
10841 measured reflections	*k* = −9→8
4602 independent reflections	*l* = −22→22

### Refinement

**Table:**

Refinement on *F*^2^	Secondary atom site location: difference Fourier map
Least-squares matrix: full	Hydrogen site location: inferred from neighbouring sites
*R*[*F*^2^ > 2σ(*F*^2^)] = 0.024	H-atom parameters constrained
*wR*(*F*^2^) = 0.057	*w* = 1/[σ^2^(*F*_o_^2^) + (0.028*P*)^2^ + 0.3633*P*] where *P* = (*F*_o_^2^ + 2*F*_c_^2^)/3
*S* = 1.09	(Δ/σ)_max_ < 0.001
4602 reflections	Δρ_max_ = 0.70 e Å^−^^3^
353 parameters	Δρ_min_ = −0.30 e Å^−^^3^
1 restraint	Absolute structure: Flack (1983), 1770 Friedel pairs
Primary atom site location: structure-invariant direct methods	Flack parameter: 0.006 (18)

### Special details

**Table:**

Geometry. All esds (except the esd in the dihedral angle between two l.s. planes) are estimated using the full covariance matrix. The cell esds are taken into account individually in the estimation of esds in distances, angles and torsion angles; correlations between esds in cell parameters are only used when they are defined by crystal symmetry. An approximate (isotropic) treatment of cell esds is used for estimating esds involving l.s. planes.
Refinement. Refinement of *F*^2^ against ALL reflections. The weighted *R*-factor *wR* and goodness of fit *S* are based on *F*^2^, conventional *R*-factors *R* are based on *F*, with *F* set to zero for negative *F*^2^. The threshold expression of *F*^2^ > σ(*F*^2^) is used only for calculating *R*-factors(gt) *etc*. and is not relevant to the choice of reflections for refinement. *R*-factors based on *F*^2^ are statistically about twice as large as those based on *F*, and *R*- factors based on ALL data will be even larger.

### Fractional atomic coordinates and isotropic or equivalent isotropic displacement parameters (Å^2^)

**Table:**

	*x*	*y*	*z*	*U*_iso_*/*U*_eq_	
Ag1	0.062198 (19)	0.74284 (3)	0.668865 (11)	0.01870 (6)	
O1	−0.0991 (2)	0.4624 (3)	0.64039 (12)	0.0199 (4)	
H1AA	−0.0950	0.3450	0.6670	0.030*	
O2	0.1744 (2)	0.5065 (3)	0.77201 (12)	0.0217 (4)	
H2AA	0.1409	0.4170	0.7790	0.033*	
N1	−0.1155 (2)	0.7669 (4)	0.55612 (13)	0.0174 (5)	
N2	0.2684 (3)	0.8371 (3)	0.75436 (14)	0.0163 (5)	
C1	−0.1281 (3)	0.9178 (4)	0.51463 (19)	0.0223 (6)	
H1A	−0.0613	1.0090	0.5354	0.027*	
C2	−0.2364 (4)	0.9449 (4)	0.44186 (18)	0.0246 (6)	
H2A	−0.2410	1.0519	0.4152	0.030*	
C3	−0.3353 (3)	0.8132 (4)	0.41024 (19)	0.0211 (6)	
H3A	−0.4080	0.8297	0.3618	0.025*	
C4	−0.3269 (3)	0.6506 (4)	0.45167 (18)	0.0170 (6)	
C5	−0.4267 (3)	0.5080 (4)	0.42261 (17)	0.0209 (6)	
H5A	−0.5013	0.5186	0.3744	0.025*	
C6	−0.4132 (3)	0.3552 (4)	0.46543 (18)	0.0214 (6)	
H6A	−0.4774	0.2614	0.4454	0.026*	
C7	−0.3036 (3)	0.3371 (4)	0.53957 (17)	0.0187 (6)	
H7A	−0.2977	0.2327	0.5683	0.022*	
C8	−0.2059 (3)	0.4719 (4)	0.56950 (16)	0.0149 (5)	
C9	−0.2140 (3)	0.6332 (4)	0.52566 (16)	0.0141 (5)	
C10	0.3161 (3)	0.9992 (4)	0.74638 (17)	0.0187 (6)	
H10A	0.2572	1.0697	0.7061	0.022*	
C11	0.4512 (3)	1.0703 (4)	0.79573 (19)	0.0222 (6)	
H11A	0.4807	1.1847	0.7878	0.027*	
C12	0.5375 (3)	0.9694 (4)	0.85498 (18)	0.0210 (6)	
H12A	0.6277	1.0140	0.8878	0.025*	
C13	0.4907 (3)	0.7962 (4)	0.86706 (17)	0.0172 (6)	
C14	0.5745 (3)	0.6843 (4)	0.92847 (17)	0.0199 (6)	
H14A	0.6655	0.7235	0.9626	0.024*	
C15	0.5225 (3)	0.5197 (4)	0.93780 (17)	0.0206 (6)	
H15A	0.5776	0.4479	0.9788	0.025*	
C16	0.3864 (3)	0.4567 (4)	0.88625 (17)	0.0177 (6)	
H16A	0.3520	0.3443	0.8937	0.021*	
C17	0.3044 (3)	0.5596 (4)	0.82523 (16)	0.0144 (5)	
C18	0.3541 (2)	0.7342 (6)	0.81437 (14)	0.0140 (4)	
O3	0.1402 (2)	−0.0140 (3)	0.92529 (13)	0.0260 (5)	
H3B	0.1405	0.0793	0.9015	0.039*	
O4	−0.4152 (2)	−0.3754 (3)	0.69128 (14)	0.0284 (5)	
O5	−0.3271 (2)	−0.5962 (3)	0.76855 (13)	0.0273 (5)	
O6	0.1239 (2)	−0.5246 (3)	0.98348 (12)	0.0211 (4)	
O7	0.1678 (2)	−0.2656 (4)	1.03550 (11)	0.0289 (4)	
O8	−0.0981 (2)	0.1498 (3)	0.70194 (12)	0.0242 (5)	
O9	0.0667 (2)	0.2136 (3)	0.81890 (12)	0.0198 (5)	
N3	−0.3217 (2)	−0.4436 (3)	0.74770 (14)	0.0173 (5)	
N4	0.1088 (2)	−0.3646 (3)	0.98082 (14)	0.0151 (5)	
C19	−0.1971 (3)	−0.3338 (4)	0.79249 (18)	0.0131 (6)	
C20	−0.1028 (3)	−0.3990 (3)	0.86303 (16)	0.0128 (5)	
H20A	−0.1152	−0.5127	0.8803	0.015*	
C21	0.0095 (3)	−0.2909 (3)	0.90660 (15)	0.0118 (6)	
C22	0.0328 (3)	−0.1188 (4)	0.88193 (16)	0.0131 (5)	
C23	−0.0605 (3)	−0.0602 (3)	0.80700 (16)	0.0130 (5)	
C24	−0.1769 (3)	−0.1677 (4)	0.76339 (18)	0.0137 (6)	
H24A	−0.2405	−0.1286	0.7152	0.016*	
C25	−0.0306 (3)	0.1145 (4)	0.77252 (16)	0.0150 (5)	

### Atomic displacement parameters (Å^2^)

**Table:**

	*U*^11^	*U*^22^	*U*^33^	*U*^12^	*U*^13^	*U*^23^
Ag1	0.01624 (8)	0.01725 (9)	0.01946 (9)	−0.00342 (12)	−0.00152 (6)	0.00022 (11)
O1	0.0213 (10)	0.0127 (9)	0.0199 (10)	−0.0063 (8)	−0.0062 (8)	0.0045 (7)
O2	0.0185 (9)	0.0169 (10)	0.0244 (11)	−0.0079 (8)	−0.0046 (8)	0.0028 (8)
N1	0.0194 (9)	0.0143 (15)	0.0179 (10)	−0.0003 (11)	0.0034 (8)	0.0009 (10)
N2	0.0138 (10)	0.0164 (12)	0.0191 (12)	−0.0018 (9)	0.0047 (9)	0.0005 (9)
C1	0.0256 (14)	0.0145 (13)	0.0262 (16)	−0.0040 (13)	0.0053 (12)	0.0045 (11)
C2	0.0331 (16)	0.0181 (15)	0.0238 (16)	0.0071 (14)	0.0092 (13)	0.0106 (12)
C3	0.0216 (13)	0.0248 (14)	0.0156 (15)	0.0061 (12)	0.0021 (11)	0.0024 (11)
C4	0.0139 (12)	0.0219 (15)	0.0152 (14)	0.0027 (11)	0.0036 (10)	0.0011 (12)
C5	0.0161 (13)	0.0293 (16)	0.0149 (14)	0.0008 (13)	−0.0007 (10)	−0.0035 (12)
C6	0.0154 (13)	0.0245 (15)	0.0215 (15)	−0.0088 (12)	−0.0008 (10)	−0.0052 (12)
C7	0.0181 (13)	0.0179 (15)	0.0188 (14)	−0.0042 (11)	0.0023 (10)	0.0014 (11)
C8	0.0142 (12)	0.0145 (13)	0.0143 (13)	0.0003 (11)	0.0002 (10)	0.0000 (10)
C9	0.0142 (12)	0.0143 (13)	0.0135 (13)	−0.0007 (11)	0.0030 (9)	0.0009 (10)
C10	0.0208 (13)	0.0172 (14)	0.0199 (15)	−0.0023 (12)	0.0082 (11)	0.0027 (11)
C11	0.0261 (14)	0.0167 (14)	0.0257 (16)	−0.0085 (13)	0.0098 (12)	−0.0039 (12)
C12	0.0182 (13)	0.0228 (15)	0.0231 (15)	−0.0110 (12)	0.0071 (11)	−0.0096 (12)
C13	0.0141 (11)	0.0225 (15)	0.0162 (13)	−0.0030 (10)	0.0064 (10)	−0.0058 (10)
C14	0.0112 (11)	0.0293 (15)	0.0177 (14)	−0.0033 (11)	0.0005 (10)	−0.0069 (10)
C15	0.0138 (12)	0.0278 (16)	0.0181 (14)	0.0033 (12)	0.0000 (10)	0.0019 (11)
C16	0.0162 (12)	0.0153 (13)	0.0207 (14)	−0.0018 (11)	0.0032 (10)	−0.0002 (10)
C17	0.0111 (11)	0.0143 (13)	0.0172 (14)	−0.0019 (11)	0.0028 (10)	−0.0028 (10)
C18	0.0113 (9)	0.0151 (11)	0.0166 (11)	0.0013 (17)	0.0052 (8)	0.0003 (15)
O3	0.0224 (10)	0.0222 (11)	0.0283 (12)	−0.0065 (9)	−0.0032 (9)	0.0033 (9)
O4	0.0209 (10)	0.0281 (12)	0.0268 (12)	−0.0043 (9)	−0.0115 (8)	−0.0011 (9)
O5	0.0294 (11)	0.0195 (11)	0.0293 (12)	−0.0131 (10)	0.0006 (9)	0.0017 (9)
O6	0.0213 (10)	0.0160 (10)	0.0235 (11)	0.0028 (8)	0.0008 (8)	0.0058 (8)
O7	0.0344 (9)	0.0225 (10)	0.0200 (9)	0.0054 (15)	−0.0118 (7)	−0.0024 (13)
O8	0.0317 (11)	0.0166 (11)	0.0195 (11)	−0.0060 (9)	−0.0027 (8)	0.0065 (8)
O9	0.0211 (8)	0.0132 (14)	0.0224 (9)	−0.0063 (9)	0.0004 (7)	0.0004 (8)
N3	0.0140 (10)	0.0190 (12)	0.0169 (12)	−0.0061 (10)	0.0004 (9)	−0.0041 (9)
N4	0.0115 (10)	0.0166 (12)	0.0153 (11)	0.0017 (9)	−0.0004 (8)	0.0023 (9)
C19	0.0078 (11)	0.0166 (13)	0.0139 (14)	−0.0039 (10)	0.0007 (10)	−0.0040 (11)
C20	0.0160 (12)	0.0084 (12)	0.0140 (13)	−0.0004 (10)	0.0034 (9)	0.0006 (9)
C21	0.0095 (9)	0.0131 (18)	0.0109 (11)	0.0044 (10)	−0.0009 (8)	0.0026 (9)
C22	0.0084 (11)	0.0151 (14)	0.0147 (13)	−0.0009 (10)	0.0011 (9)	−0.0027 (10)
C23	0.0130 (11)	0.0110 (13)	0.0139 (12)	−0.0003 (11)	0.0013 (9)	−0.0003 (10)
C24	0.0122 (12)	0.0145 (14)	0.0142 (14)	0.0023 (11)	0.0028 (10)	0.0010 (11)
C25	0.0151 (12)	0.0110 (12)	0.0182 (14)	0.0005 (11)	0.0030 (10)	0.0009 (10)

### Geometric parameters (Å, °)

**Table:**

Ag1—N2	2.183 (2)	C12—C13	1.415 (4)
Ag1—N1	2.190 (2)	C12—H12A	0.9300
Ag1—O2	2.549 (2)	C13—C18	1.415 (4)
Ag1—O1	2.561 (2)	C13—C14	1.417 (4)
O1—C8	1.352 (3)	C14—C15	1.361 (4)
O1—H1AA	0.9999	C14—H14A	0.9300
O2—C17	1.355 (3)	C15—C16	1.406 (4)
O2—H2AA	0.7666	C15—H15A	0.9300
N1—C1	1.341 (4)	C16—C17	1.367 (4)
N1—C9	1.366 (4)	C16—H16A	0.9300
N2—C10	1.325 (4)	C17—C18	1.430 (5)
N2—C18	1.368 (4)	O3—C22	1.330 (3)
C1—C2	1.395 (4)	O3—H3B	0.8193
C1—H1A	0.9300	O4—N3	1.229 (3)
C2—C3	1.361 (5)	O5—N3	1.220 (3)
C2—H2A	0.9300	O6—N4	1.225 (3)
C3—C4	1.420 (4)	O7—N4	1.217 (3)
C3—H3A	0.9300	O8—C25	1.241 (3)
C4—C5	1.419 (4)	O9—C25	1.274 (3)
C4—C9	1.422 (4)	N3—C19	1.457 (3)
C5—C6	1.364 (4)	N4—C21	1.472 (3)
C5—H5A	0.9300	C19—C20	1.386 (4)
C6—C7	1.409 (4)	C19—C24	1.387 (4)
C6—H6A	0.9300	C20—C21	1.373 (4)
C7—C8	1.366 (4)	C20—H20A	0.9300
C7—H7A	0.9300	C21—C22	1.409 (4)
C8—C9	1.432 (4)	C22—C23	1.421 (4)
C10—C11	1.407 (4)	C23—C24	1.393 (4)
C10—H10A	0.9300	C23—C25	1.507 (4)
C11—C12	1.354 (4)	C24—H24A	0.9300
C11—H11A	0.9300		
			
N2—Ag1—N1	151.54 (9)	C10—C11—H11A	120.6
N2—Ag1—O2	68.97 (8)	C11—C12—C13	120.1 (3)
N1—Ag1—O2	138.45 (9)	C11—C12—H12A	119.9
N2—Ag1—O1	138.63 (8)	C13—C12—H12A	119.9
N1—Ag1—O1	69.23 (8)	C18—C13—C12	117.5 (3)
O2—Ag1—O1	69.67 (6)	C18—C13—C14	119.5 (3)
C8—O1—Ag1	111.74 (16)	C12—C13—C14	123.0 (3)
C8—O1—H1AA	113.3	C15—C14—C13	120.3 (2)
Ag1—O1—H1AA	134.7	C15—C14—H14A	119.8
C17—O2—Ag1	112.59 (16)	C13—C14—H14A	119.8
C17—O2—H2AA	117.9	C14—C15—C16	120.8 (3)
Ag1—O2—H2AA	129.2	C14—C15—H15A	119.6
C1—N1—C9	118.3 (2)	C16—C15—H15A	119.6
C1—N1—Ag1	119.1 (2)	C17—C16—C15	120.5 (3)
C9—N1—Ag1	122.58 (19)	C17—C16—H16A	119.7
C10—N2—C18	118.3 (3)	C15—C16—H16A	119.7
C10—N2—Ag1	118.73 (19)	O2—C17—C16	123.9 (3)
C18—N2—Ag1	123.0 (2)	O2—C17—C18	115.9 (2)
N1—C1—C2	123.3 (3)	C16—C17—C18	120.3 (2)
N1—C1—H1A	118.4	N2—C18—C13	121.9 (3)
C2—C1—H1A	118.4	N2—C18—C17	119.6 (2)
C3—C2—C1	119.4 (3)	C13—C18—C17	118.6 (3)
C3—C2—H2A	120.3	C22—O3—H3B	109.5
C1—C2—H2A	120.3	O5—N3—O4	124.3 (2)
C2—C3—C4	119.6 (3)	O5—N3—C19	118.3 (2)
C2—C3—H3A	120.2	O4—N3—C19	117.4 (2)
C4—C3—H3A	120.2	O7—N4—O6	124.3 (2)
C5—C4—C3	122.8 (3)	O7—N4—C21	119.0 (2)
C5—C4—C9	119.6 (3)	O6—N4—C21	116.7 (2)
C3—C4—C9	117.7 (3)	C20—C19—C24	122.2 (3)
C6—C5—C4	119.9 (3)	C20—C19—N3	118.7 (3)
C6—C5—H5A	120.0	C24—C19—N3	119.1 (3)
C4—C5—H5A	120.0	C21—C20—C19	118.0 (2)
C5—C6—C7	121.2 (3)	C21—C20—H20A	121.0
C5—C6—H6A	119.4	C19—C20—H20A	121.0
C7—C6—H6A	119.4	C20—C21—C22	122.6 (2)
C8—C7—C6	120.5 (3)	C20—C21—N4	116.7 (2)
C8—C7—H7A	119.8	C22—C21—N4	120.7 (2)
C6—C7—H7A	119.8	O3—C22—C21	122.2 (2)
O1—C8—C7	123.1 (2)	O3—C22—C23	120.1 (3)
O1—C8—C9	116.7 (2)	C21—C22—C23	117.7 (2)
C7—C8—C9	120.2 (2)	C24—C23—C22	119.9 (3)
N1—C9—C4	121.7 (2)	C24—C23—C25	119.5 (2)
N1—C9—C8	119.7 (2)	C22—C23—C25	120.5 (2)
C4—C9—C8	118.6 (2)	C19—C24—C23	119.4 (3)
N2—C10—C11	123.4 (3)	C19—C24—H24A	120.3
N2—C10—H10A	118.3	C23—C24—H24A	120.3
C11—C10—H10A	118.3	O8—C25—O9	125.1 (3)
C12—C11—C10	118.9 (3)	O8—C25—C23	118.7 (2)
C12—C11—H11A	120.6	O9—C25—C23	116.1 (2)

### Hydrogen-bond geometry (Å, °)

**Table:**

*D*—H···*A*	*D*—H	H···*A*	*D*···*A*	*D*—H···*A*
O1—H1AA···O8	1.00	1.60	2.602 (3)	175
O2—H2AA···O9	0.77	1.88	2.636 (3)	168
O3—H3B···O9	0.82	1.74	2.483 (3)	150
